# High failure rate of proximal femoral locking plates in fixation of trochanteric fractures

**DOI:** 10.1186/s13018-018-0951-6

**Published:** 2018-10-05

**Authors:** Shuangjian He, Bin Yan, Jian Zhu, Xiaoyi Huang, Jianning Zhao

**Affiliations:** 1 Department of Orthopaedics, Taixing People’s Hospital, Taixing, Jiangsu People’s Republic of China; 2 Department of Orthopaedics, Jinling Clinical Medical College, Nanjing Medical University, Nanjing, Jiangsu China; 3 Department of Orthopaedics, Jinling Hospital, Nanjing Medical University, No. 305, Zhongshan East Road, Nanjing, 210002 Jiangsu China

**Keywords:** Trochanteric fractures, Hip fracture, Locking plate, Mechanical failure, Complications

## Abstract

**Background:**

The aim of this study was to report our previous results of treatments for trochanteric fractures with proximal femoral locking plates (PFLP) and to analyze the underlying mechanisms and possible risk factors associated with the high failure rate of this technique.

**Methods:**

From January 2010 to October 2014, 273 consecutive patients with trochanteric femoral fractures were identified, and 95 patients (with 97 fractures) ultimately met the inclusion criteria. Clinical records regarding demographic features and intraoperative data including total incision length, operation time, blood loss, and failures detected in radiographs were documented and assessed. The collected data were analyzed with SPSS 19.0 software.

**Results:**

The stable group (AO/OTA 31 A1 and A2.1) had less blood loss than the unstable group (AO/OTA 31 A2.2, A2.3, and A3). The ultimate failure rate was 36% in 97 fractures. The obvious complications in this study included nonunion in 7 (7.2%) fractures, implant breakage in 4 (4.1%) fractures, varus deformity in 34 (35%) fractures, and loosening of the proximal femoral screw in 21 (21.6%) fractures. Six patients received reoperations. The total failure rate in the stable group was 17% and was 50% in the unstable group. In patients greater than 60 years old in the unstable group, the failure rate was 60.5%.

**Conclusions:**

High failure rates of PFLP were observed in patients with trochanteric fracture, especially in patients who were greater than 60 years old with unstable fracture types. PFLP was not an appropriate treatment for trochanteric fractures.

## Background

With the increase in the aging population, trochanteric fractures including pertrochanteric, intertrochanteric, and subtrochanteric fractures also have a rising trend, and most of these fractures need surgical treatment [1–4]. For stable fracture types, either extramedullary or intramedullary implants such as the dynamic hip screw (DHS), the dynamic condylar screw (DCS), and proximal femoral nail anti-rotation (PFNA) are considered to be successful devices [1, 5]. According to published studies, however, the most effective implant for the treatment of unstable trochanteric fractures is still being debated [6–11]. In most previous studies, intramedullary devices were recommended for patients with unstable fractures patterns and reportedly achieved better clinical results with lower complications than extramedullary implants. However, some authors suggested that the use of intramedullary devices had no significant advantage over extramedullary devices, especially in cases with highly comminuted fractures at the site of nail insertion and the lateral femoral wall both of which are considered major risks related to higher failure rates [12, 13].

As an extramedullary device, the proximal femoral locking plates (PFLP) has the advantage of angular stable fixation, and it can preserve more bone stock. The PFLP is considered an alternative fixation method for most complex proximal femoral fractures and even led to excellent results for management of unstable fractures [9, 10, 14, 15]. However, some studies have drawn attention to the higher than expected failure rates of PFLPs [11, 16, 17]. Unfortunately, few clinical data of case series are available to evaluate the use of PFLPs. The purpose of this study was to retrospectively report our previous results for trochanteric fractures treated with PFLPs and to analyze the underlying mechanisms as well as possible risk factors associated with the high failure rate of this technique.

## Methods

### Ethics statement

All clinical records and radiological data for this retrospective cohort study were approved by the ethics committee. Informed consent was obtained from all the patients.

### Patient population and data collection

From January 2010 to October 2014, 273 consecutive patients with trochanteric femoral fractures who received a PFLP (5.0/6.0 Shanghai PuWei Medical Device Factory Co.) in our institutional orthopedic trauma center were identified. The inclusion criteria were the presence of pertrochanteric, intertrochanteric, or subtrochanteric fractures. Patients with pathological fractures (other than osteoporosis), previous fractures, open fractures, combined fractures on the ipsilateral side; patients with less than 12 months of follow-up; and patients with consecutive postoperative radiograph were excluded from this study.

All operations were performed by experienced surgeons who received training for using PFLPs. This plate has three proximal holes angled at 115° for 6.0 mm locking screw fixation into the femoral neck and head, and the remaining distal holes were inserted with either 4.5 mm nonlocking cortex screws or 5.0 mm locking screws to obtain femoral shaft fixation. Generally, a lateral subvastus approach to the proximal femur for open reduction and internal fixation was used for all cases. After the operation, partial and progressive weight bearing was encouraged based on how the callus formed on the radiograph.

Clinical data collected included patient age, gender, laterality, mechanism of injury, fracture pattern, time from fracture to surgery, total incision length, operation time, blood loss, revision procedure, and other data. Fractures were classified according to the AO/OTA (Orthopaedic Trauma Association) classification system. The stable fracture was defined as type of AO/OTA 31 A1 and A2.1, and the unstable fracture was defined as type of AO/OTA 31 A2.2, A2.3 and A3. The first postoperative radiograph and each follow-up anteroposterior and lateral radiographs were reviewed to assess fracture type, reduction status, screw position, neck-shaft angle, callus formation, and device failure.

Mechanical failure was defined as breakage of the implant, loosening of the proximal screw, varus deformity of the fracture, secondary loss of reduction, and shortening of the femoral neck. Additionally, nonunion and reoperations were quantified. Bone union was defined as the disappearance of the fracture line or radiological evidence of callus formation with no tenderness.

### Statistical methods

All data were analyzed using SPSS 19.0 software. Chi-squared tests (continuity correction or linear-by-linear association) were used for comparison of categorical variables. Continuous variables were compared using an independent *t* test and one-way ANOVA. The difference between the groups was considered to be statistically significant when *p* < 0.05 in a two-sided test.

## Results

A total of 95 patients (58 males, 37 females; mean age 66.8 years, range 32–92 years) met the inclusion criteria and ultimately served as the reviewed study group. The causes of injury included a ground-level slip in 64 patients (67%), fall from a height in 22 patients (23%), and traffic accident in 9 patients (10%). Of these 95 patients, 2 patients had bilateral side trochanteric fractures, which resulted in 97 fractures that were classified as AO types (31 A1.1 = 4, A1.2 = 15, A1.3 = 4, A2.1 = 18, A2.2 = 17, A2.3 = 7, A3.1 = 2, A3.2 = 3, A3.3 = 27) (Table 1). Of the 97 fractures, 51 (53%) fractures involved the right side, and 46 (47%) fractures involved the left side. The mean time from fracture to surgery was 4.3 days (range 1–11). The mean total incision length was 16.6 cm (range 12–30). The mean operation time (from the beginning of the skin incision to the closure of wound) was 131.5 min (range 60–230), and the mean blood loss was 477.7 ml (range 200–1500) (Table 1).

**Table 1:** Perioperative variables plate

	Included (*n* = 95 patients/97 fractures)		
	*n* (%)	Mean (±SD)	Range
Number of patients	95		
Number of fractures	97		
Age (years)*		66.8 ± 14.7	32–92
< 60*	21 (22)		
60 or older*	74 (78)		
Gender*			
Male*	58 (61)		
Female*	37 (39)		
Mechanism of injury			
Ground level fall*	64 (67)		
Fall from a height*	22 (23)		
Traffic accident*	9 (10)		
AO fracture types†			
A1.1†	4 (4)		
A1.2†	15 (15)		
A1.3†	4 (4)		
A2.1†	18 (19)		
A2.2†	17 (18)		
A2.3†	7 (7)		
A3.1†	2 (2)		
A3.2†	3 (3)		
A3.3†	27 (29)		
Laterality†			
Left†	46 (47)		
Right†	51 (53)		
Time from fracture to surgery (days)		4.3 ± 2.1	1–11
Total incision length (cm)		16.6 ± 3.1	12–30
Operative time (min)		131.5 ± 38.9	60–230
Blood loss (ml)		477.7 ± 202.5	200–1500
Total failure of fractures†	35 (36)		
Breakage of implant†	4 (4)		
Loosening of proximal screw†	21 (22)		
Varus deformity†	34 (35)		
Nonunion†	7 (7)		
Reoperation†	6 (6)		

Quantitative data were presented as mean (SD)

*For included patients, values based on number of patients (*n* = 61 no failure, *n* = 34 failure)

†Values based on number of fractures (*n* = 62 no failure, *n* = 35 failure)

Of the 95 patients (97 fractures) in this study, 21 (22%) patients were younger than 60 years old, and 74 (78%) patients were 60 years old or older. The stable group had less blood loss compared to the unstable group (403 ± 101 vs. 508 ± 218 ml; *p* = 0.014), while the mean incision length and the operation time between two groups had no statistically significant difference (*p* > 0.05). The variables of the two groups are shown in Table 2.

**Table 2:** Perioperative data in relation to fracture type

	Stable	Unstable	Total	*p* value
	A1.1-A2.1	A2.2-A3.3		
Number of fractures	41 (42%)	56 (58%)	97	
Mean operative time (min)	117 (39)	129 (35)	125 (37)	0.172*
Mean intraoperative blood loss (ml)	403 (101)	508 (218)	466 (187)	0.014*
Mean operative incision length (cm)	16 (2.1)	17 (3.4)	16.6 (3)	0.108*

Quantitative data were presented as mean (SD)

*Analyzed using a one-way ANOVA

**Fig. 1:**
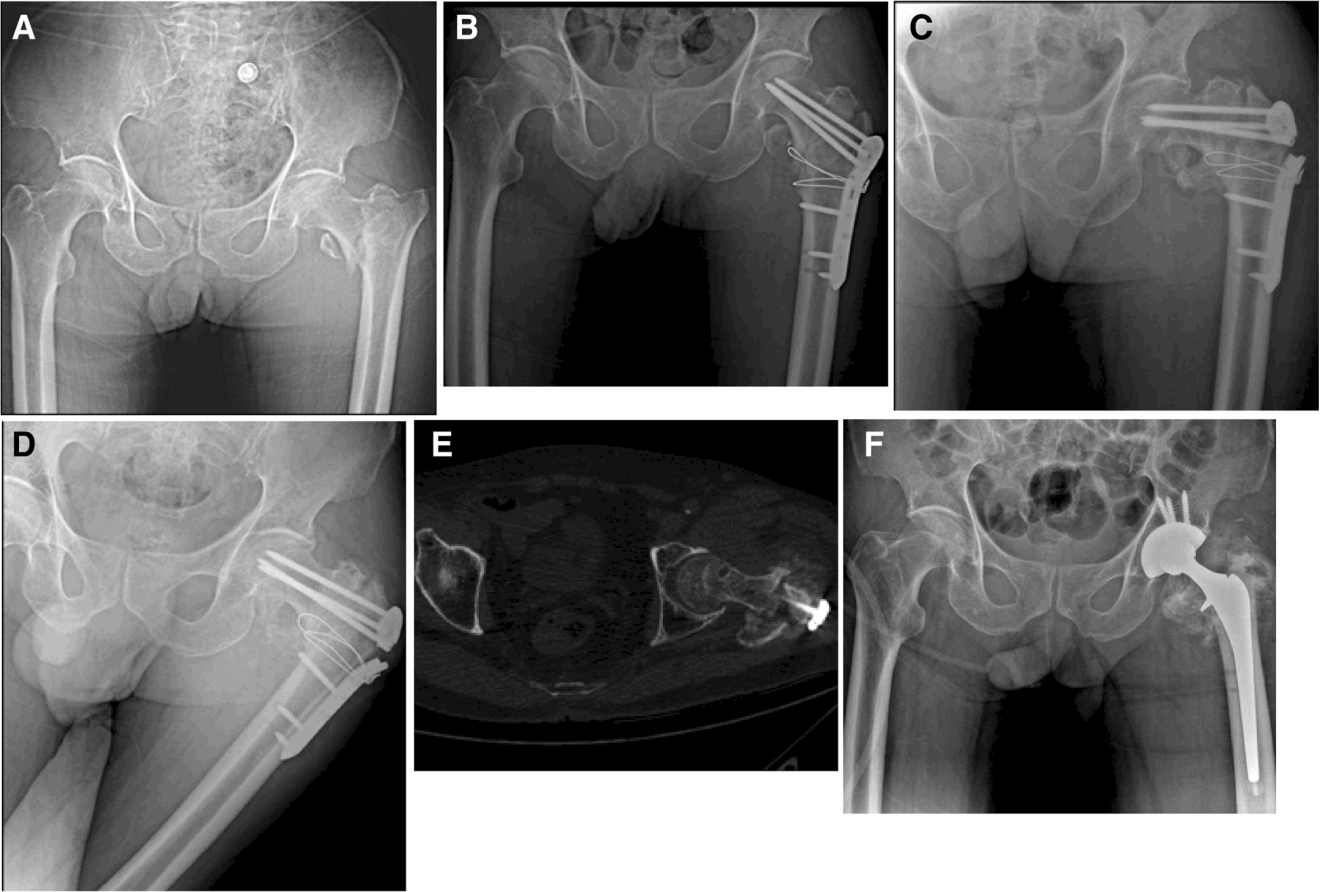
A 75-year-old male with AO/OTA type 31A2.2 experienced plate breakage. **a** Radiograph after injury. **b** Postoperative radiograph showing good reduction and fixation with PFLP (1 week after surgery). **c** Plate breakage and varus collapse 16 weeks after surgery. **d**, **e** Postoperative radiograph and computed tomography (CT) showing nonunion (18 weeks after surgery). **f** Revision surgery of THA

**Fig. 2:**
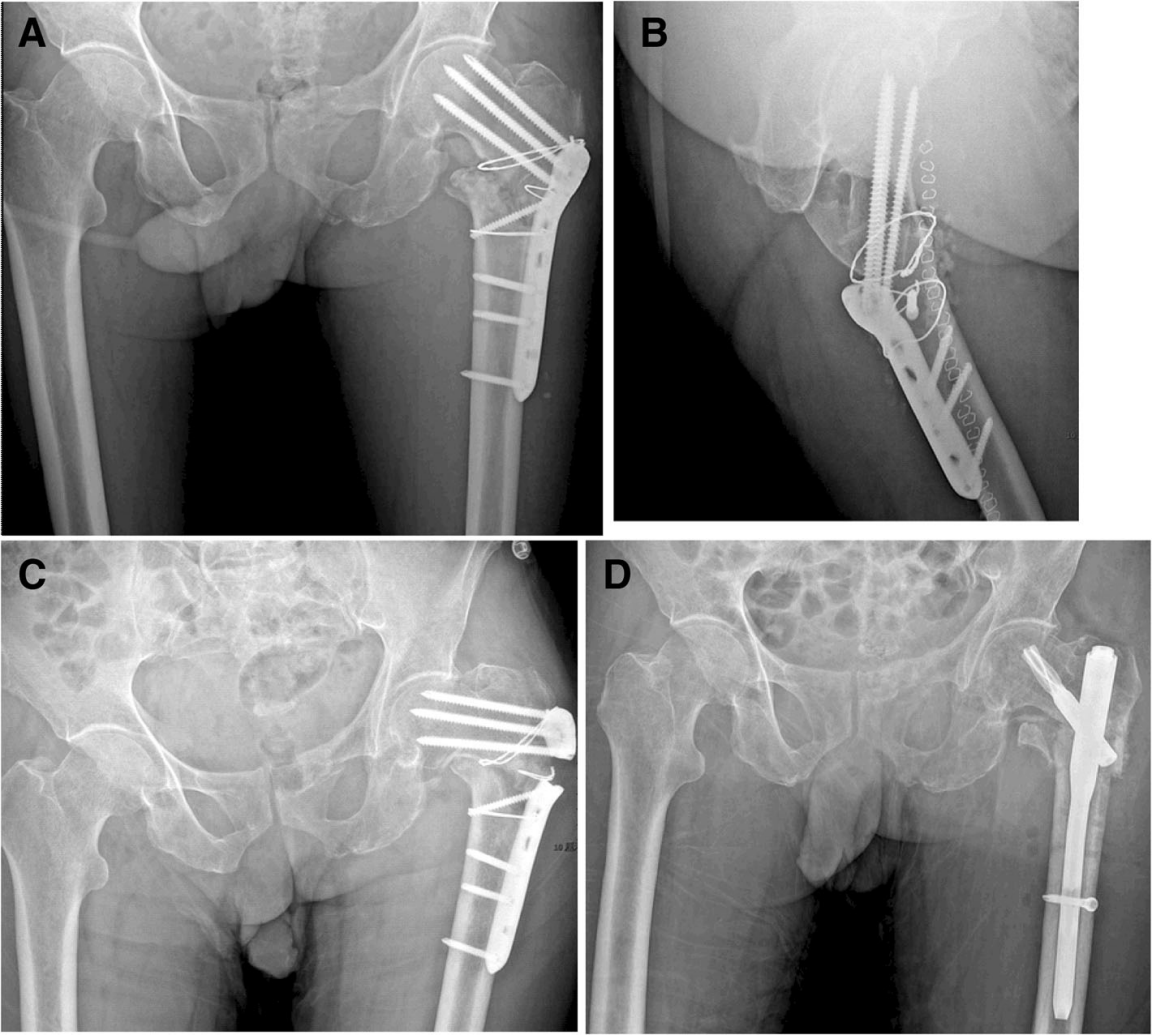
A 78-year-old male with AO/OTA type 31A3.3 experienced plate breakage. **a**, **b** Postoperative anterio-posterior and lateral radiographs. **c** Plate breakage and varus collapse 10 weeks after surgery. **d** Postoperative radiograph of revision surgery of PFNA

**Fig. 3:**
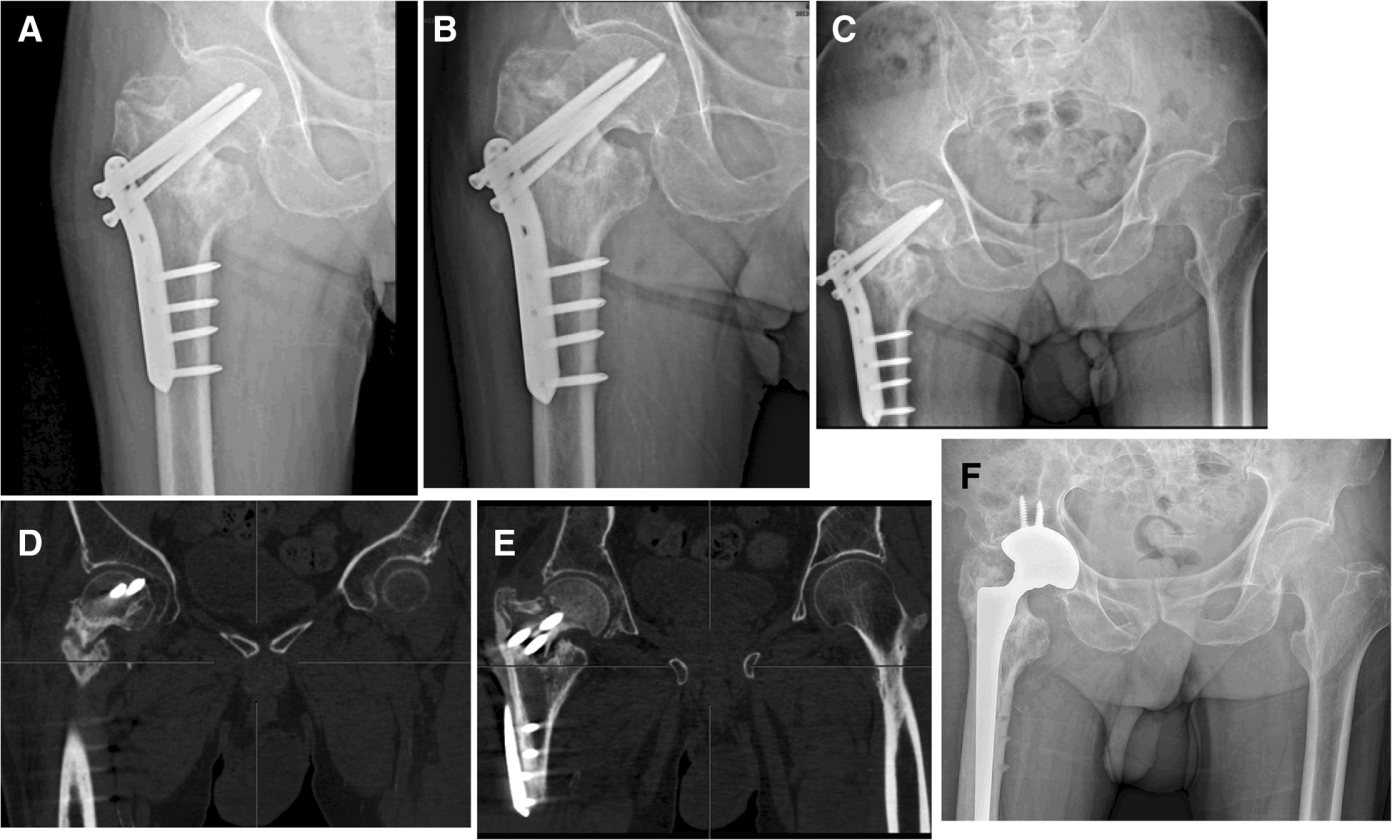
A 79-year-old male with AO/OTA type 31A2.3 suffered mechanical failure and nonunion. **a** Postoperative radiograph showing loosening of proximal screws and varus collapse 36 weeks after surgery. **b**, **c** Postoperative radiograph showing that progressive loosening and penetration through femoral head of proximal femoral screws as well as shortening of femoral neck (44 and 56 weeks after surgery, respectively). **d**, **e** CT scan showing nonunion and penetration through femoral head (56 weeks after surgery). **f** Revision surgery of THA

**Fig. 4:**
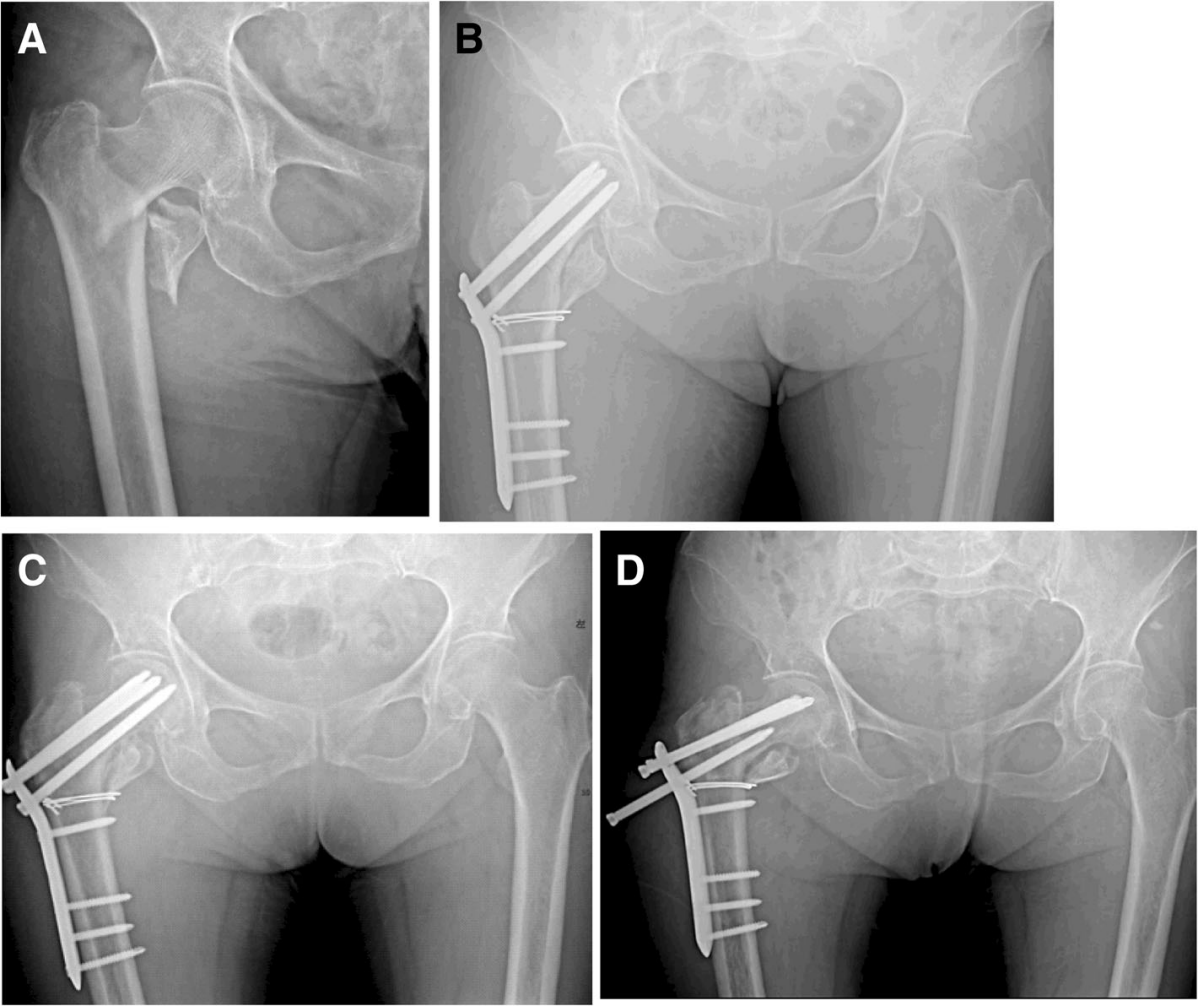
A 81-year-old female with AO/OTA type 31A2.1 experienced loosening of proximal screws. **a** Radiograph after injury. **b** Postoperative radiograph showing good reduction and fixation (1 week after surgery). **c** Ten weeks postoperative radiograph showing loosening of proximal femoral screws, loss of reduction and varus collapse. **d** Progressive loss of reduction, screws loosening and varus collapse (24 weeks after surgery)

**Fig. 5:**
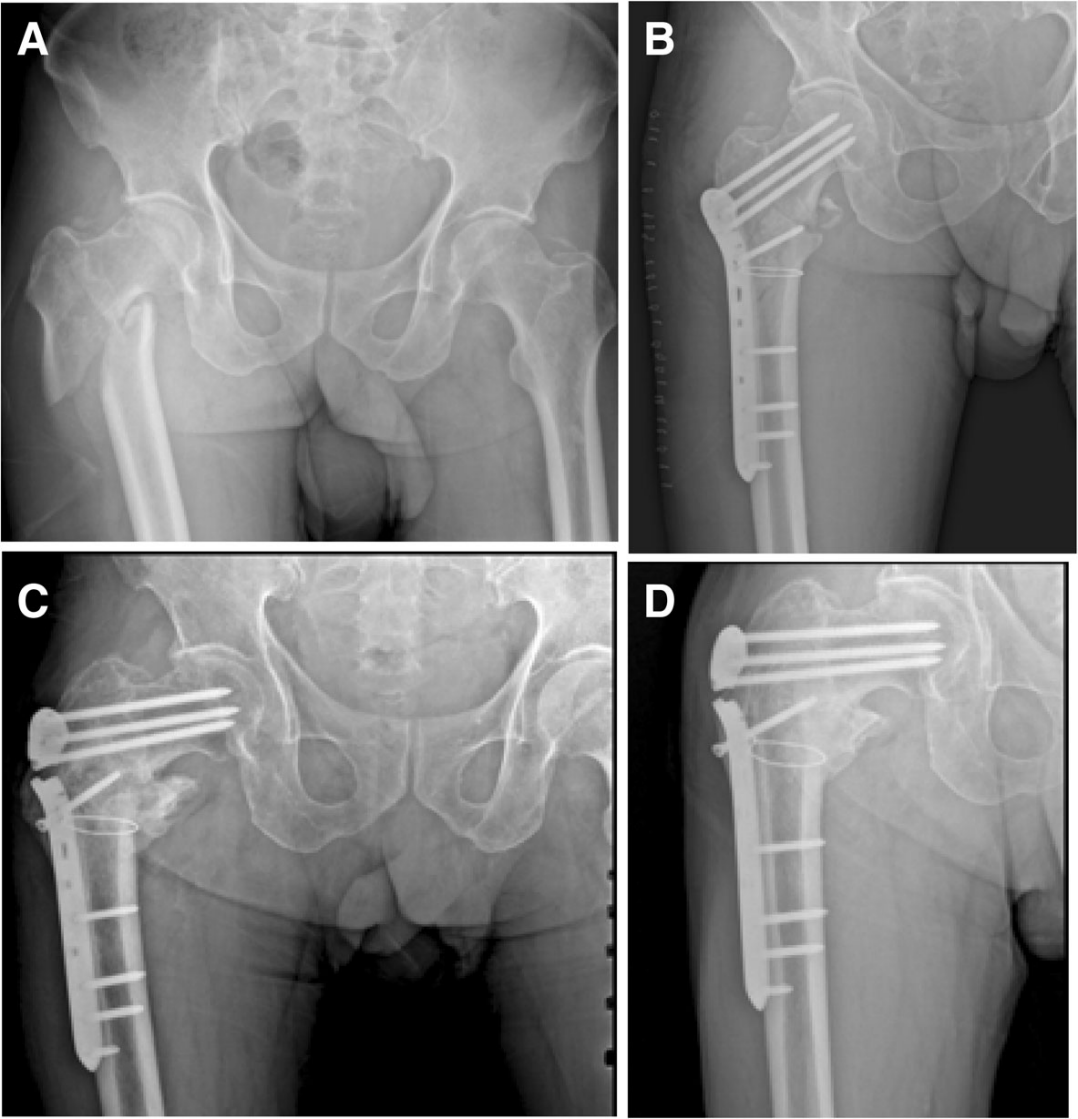
A 71-year-old male with AO/OTA type 31A3.3 experienced plate breakage. **a** Radiograph after injury. **b** Postoperative radiograph showing good reduction and fixation with PFLP (1 week after surgery). **c** Plate breakage and varus collapse 36 weeks after surgery. **d** Seventy-two weeks postoperative radiograph showing malunion and varus deformity

Among 95 patients (97 fractures), 34 patients (35 fractures) suffered operation failure, which ultimately presented a high failure rate of 36%. The complications in this study included nonunion in 7 (7.2%) fractures, implant breakage in 4 (4.1%) fractures, varus deformity in 34 (35%) fractures, and loosening of proximal femoral screw in 21 (21.6%) fractures (Table 3; Figs. 1, 2, 3, 4, and 5).

**Table 3:** Postoperative mechanical failure in relation to fracture type

	Included (*n* = 95 patients/97 fractures)			*p* value
	Stable*	Unstable*	Total*	
	*n* (%)	*n* (%)	*n* (%)	
Number of fractures	41 (42)	56 (58)	97 (100)	
Nonunion	0 (0)	7 (12.5)	7 (7.2)	0.02†
Breakage of implant	0 (0)	4 (7.1)	4 (4.1)	0.135†
Varus deformity	6 (14.6)	28 (50)	34 (35)	0.001†
Loosening of proximal screw	4 (9.7)	17 (30.3)	21 (21.6)	0.023†
Total failure rate	7 (17)	28 (50)	35 (36)	

*Values based on number of fractures (*n* = 62 no failure, *n* = 35 failure)

†Analyzed using an *x*^2^ test (continuity correction)

In the 97 fractures included in this study, 41 (42%) were stable type fractures and 56 (58%) were unstable type fractures. The failure rates in relation to fractures types among groups of A1, A2, and A3 fractures were 13%, 38%, and 50%, respectively. The failure rates for nonunion implant breakage, varus deformity, and screw loosening in the stable group were 0%, 0%, 14.6%, and 9.7%, respectively, and the failure rates for patients in the unstable groups were 12.5%, 7.1%, 50%, and 30.3%, respectively. The total failure rate in the stable group was 17% compared to 50% in the unstable group (Table 3).

The total failure rates and the rates of nonunion, implant breakage, varus deformity, and screw loosening in patients older than 60 years old were significantly higher compared to patients less than 60 years old (44.5% vs. 9.5%; 9.5% vs. 0%; 5.4% vs. 0%; 43% vs. 9.5% and 27% vs. 0.5%, respectively). The variables for the two groups are shown in Table 4. Furthermore, the failure rate of PFLP in patients older than 60 years old with unstable fracture types was 60.5% (Table 5).

**Table 4:** Mechanical failure rate in relation to age of patients

	Age less than 60	60 or older	Total
Number of patients	21 (22%)	74 (78%)	95 (100%)
Number of patients with failure (%)	2 (9.5)	33 (44.5)	35
Nonunion (%)†	0 (0)	7 (9.5)	7
Breakage of implant (%)†	0 (0)	4 (5.4)	4
Varus deformity (%)†	2 (9.5)	32 (43)	34
Loosening of proximal screw (%)†	1 (0.5)	20 (27)	21

†Values based on number of patients with fractures (*n* = 21 less than 60 years, *n* = 74 more than 60 years)

**Table 5:** Failure rates and reoperation in patients older than 60 years with unstable fracture types

Variables	Value (*n*)	Percentage (%)
Patients older than 60 years with unstable fractures	43	100%
Number of patients with failure	26	60.5%
Nonunion†	6	13.9%
Breakage of implant†	4	9.3%
Varus deformity (%)†	26	60.5%
Loosening of proximal screw †	16	37.3%
Number of reoperation	6	13.9%

Six patients received revision operations, including reoperation with total hip arthroplasty (THA) in three patients, fixation of the PFNA in two patients, and a Gamma-nail procedure in one patient (Table 6).

**Table 6:** Data of reoperation cases

Case	Gender	Age (years)	Fracture type	Failure mode	Revision
1	M	61	A2.2	Severe varus collapse	THA
2	M	79	A2.3	Varus collapse, screw loosening, nonunion	THA
3	M	75	A2.2	Nonunion, breakage of implant	THA
4	M	78	A3.3	Nonunion, breakage of implant	PFNA
5	M	75	A3.3	Varus collapse, screw loosening, nonunion	PFNA
6	F	69	A3.3	Severe varus collapse, screw loosening;	Gamma-nail

## Discussion

As an alternative implant for extramedullary devices, PFLP has become increasingly popular due to its advantage in proximal multiple angle-stable screws, which can enhance proximal femoral fixation and preserve more bone stock by leaving a smaller “footprint” after placement than other extramedullary plates with large proximal screws [18]. Furthermore, previous studies have shown that PFLP presented with equivalent biomechanical properties as other angularly stable implants or intramedullary nails [19–21]. Owing to biomechanical peculiarities, PFLP fixation has been recommended for fixation of complex proximal femoral fractures, such as osteoporotic, comminuted, or unstable fractures as well as for revision fixation [22]. A series of studies have reported that the fixation with PFLP in cases of unstable trochanteric fractures can achieve satisfying radiological and clinical results with a higher union rate and fewer complications [9, 23]. Naiyer et al. demonstrated that for 25 patients with unstable intertrochanteric fractures treated with a proximal femoral locking compression plate, the failure rate was 16%, which was lower than the failure rate of 51% in the DHS group of 35 patients with the same type of fracture. However, recent studies have paid attention to the higher failure rate of PFLPs, especially in cases with unstable trochanteric fractures. Philipp et al. reported that in patients with unstable 31 A3 trochanteric fractures treated with a PFLP, incidences of reoperation (25%), mechanical failure (38%), and nonunion (19%) were observed whereas these percentages were 5%, 5%, and 5% in patients treated with cephalomedullary nailing (CMN) [12]. Similarly, Streubel et al. reported a total failure rate of 33% at the 12-month follow-up point in the presence of varus collapse with proximal screw loosening, screw “cutout,” screw breakage, and plate fracture. Additionally, Wirtz et al. demonstrated the early results of PFLPs in the management of 19 patients with stable and unstable trochanteric fractures, and 8 (42%) revision surgeries were required, including reosteosynthesis and THA because of secondary loss of reduction or implant removal [17].

In the present study, the cumulative failure rates of mechanical failure and nonunion in a consecutive cohort of patients with trochanteric fractures (AO/OTA type 31A1-A3) treated with PFLP, were reported. Furthermore, difference in intraoperative data, postoperative complications, and reoperations in relation to the patient’s age and fracture types were analyzed to investigate the possible risk factors and underlying mechanisms associated with high failure rates when using this technique.

The overall failure rate was proximally 36% in 95 patients with 97 fractures after 12 months of follow-up. There was no difference in the number of complications according to gender and injury mechanism. The most frequent failure was varus deformity with a 35% failure rate, followed by loosening of the proximal screw with a failure rate of 22%. Based on prior studies and our present results, several factors seem to have played an important role in relation to high failure rates. First, the age of the patients may influence surgical outcomes. In the present study, the failure rate in patients 60 years old or older was 44.5%, which was significantly higher than the 9.5% of patients who were younger than 60 years old. Among these observed failures, varus deformity (43%) and loosening of proximal screw (27%) in the elderly group were more likely to occur, which was probably due to the weakness of holding power resulting from poor bone quality especially in elderly patients with osteoporosis. Second, the type of fracture also make a great contribution to the failure. In our study, the failure rates among the groups with A1, A2, and A3 fractures were 13%, 38%, and 50%, respectively. The failure rate in the unstable group reached up to 50%, which was markedly higher than the 17% failure rate in the stable group. Additionally, in patients older than 60 years old with unstable fractures, the failure rate was as high as 60.5%, which was sufficient to suggest that the older patients who suffered unstable trochanteric fractures seemed to be the most important factor leading to a high failure rate. Third, the surgical technique, such as appropriate reduction and accurate placement of proximal screws as well as using a minimally invasive technique is also beneficial for reducing the incidence of failure. Ihab et al. compared the intraoperative differences and clinical outcomes between direct (open) reduction and indirect (biological) reduction groups with trochanteric fractures, and patients in the open group had a greater blood loss, longer operation time, and incision lengths. However, there was no difference in the healing rate or functional outcomes. In addition, closed reduction of unstable comminuted trochanteric fractures made it difficult to maintain sufficient reduction of the postero-medial region, which was one of the keys to avoiding mechanical failure. Although increasing clinical and biomechanical research has addressed the importance of the posteromedial buttress of the proximal femur, the results of other studies suggested that there was no robust evidence to confirm that the lower failure rate was associated with sufficient anatomical reduction of the medial buttress [10, 12, 17, 19, 24]. Similar results were observed in our studies in which most failure cases achieved good reduction of the medial buttress. However, in the present study, the mean intraoperative blood loss was statistically significantly lower in the stable group (*p* = 0.014). Furthermore, factors such as accurate placement of proximal screws and their appropriate position in the femoral head might also contribute to enhancing the construct strength [12]. Previous biomechanical studies have shown that a screw deviation of 2° or more from the nominal locking axis angulations with the plate would significantly reduce the stiffness and fixation stability of the screw-plate constructs, resulting in early screw loosening, progressive varus of the fracture, and even implant breakage. Therefore, accurate placement of the proximal femoral locking screws was crucial for maintaining the stable and stiff biomechanical peculiarity of this device. Finally, the design features of PFLP affect the clinical effectiveness. It was generally acknowledged that the integrity of the lateral trochanteric wall was an important factor for maintaining the stability of the proximal femoral fractures and could greatly decrease the rate of malunion or nonunion. The locking screws of the PFLP could hold all the major proximal femoral fragments due to the angular and stable design. Therefore, several studies had suggested that it was useful to apply PFLP for treating trochanteric fractures of AO/OTA type 31A3, which was usually represented by comminuted fractures of the lateral wall that might lead to surgical failure due to secondary fracture or displacement of the proximal lateral fragments when using intramedullary nails or DHS. However, other studies had claimed that the weakness of high concentrations of stress at the junction of the PFLP and the proximal locking screws, as well as the small number and size of the proximal screws, was insufficient to resist cyclic axial or torsion loading and provide stable fixation to the proximal fragment, which likely resulted in hardware failure [24]. In order to reduce the risk of mechanical failure, avoiding early weight-bearing after treatment with PFLP was recommended by many researchers. In addition, increasing the size of the screws and providing a poly-axial position for the proximal locking screws might provide more stability for the proximal fragment [17].

There are several limitations to this study, including the lack of a control group treated with other methods such as PFNA, DHS, or percutaneous compression plate (PCCP) fixation. Another drawback is the relatively small number of cases. Our group of patients (*n* = 95) may be small to achieve sufficient statistical relevance. To the best of our knowledge, however, to date, it seems to be one of the largest groups with clinical data on the results of fixation with a PFLP. In addition, this study did not include long-term follow-up of functional outcomes. Further studies should focus on the investigation of functional results from the PFLP and compare them to PFNA.

## Conclusions

Our study revealed that PFLP resulted in high failure rate of trochanteric fractures, especially in patients older than 60 years old with unstable fracture types. PFLP was not an appropriate treatment for trochanteric fractures. However, we can still use it in stable trochanteric fractures with only two fragments. Advanced age and an unstable fracture type were major risk factors for the unsatisfactory outcomes of PFLP fixation.

## Abbreviations

CMN: Cephalomedullary nailing
DCS: Dynamic condylar screw
DHS: Dynamic hip screw
PCCP: Percutaneous compression plate
PFLP: Proximal femoral locking plates
PFNA: Proximal femoral nail anti-rotation
